# Attaining functional levels of visual acuity after vitrectomy for retinal detachment secondary to proliferative diabetic retinopathy

**DOI:** 10.1038/s41598-020-72618-y

**Published:** 2020-09-24

**Authors:** Aaron Ricca, Kiley Boone, H. Culver Boldt, Karen M. Gehrs, Stephen R. Russell, James C. Folk, M. Bridget Zimmerman, Mark E. Wilkinson, Elliott H. Sohn

**Affiliations:** 1 Department of Ophthalmology and Visual Sciences, University of Iowa Hospitals and Clinics, 200 Hawkins Drive, Iowa City, IA 52242 USA; 2 Carver College of Medicine, University of Iowa, Iowa City, IA USA; 3 Department of Biostatistics, University of Iowa, Iowa City, IA USA

**Keywords:** Diabetes complications, Retinal diseases, Vision disorders

## Abstract

Most patients needing diabetic tractional retinal detachment (TRD) surgery are working-age adults that drive and participate in other vision-dependent activities of daily living. We sought to determine the proportion of patients that achieve functional visual acuity (VA) based on the World Health Organization (WHO) definition of ‘low vision’ (≤ 20/80) and US driving standards (≥ 20/40) after vitrectomy for diabetic TRD. In this 10-year retrospective review, consecutive patients who underwent primary vitrectomy for TRD from proliferative diabetic retinopathy were studied. 240 eyes in 203 patients met criteria for analysis (38 eyes were lost to follow up at 3 months; 68 at 12 months; 146 at 60 months). While most patients (nearly 80%) having TRD surgery had low vision pre-op, almost half attained VA that was > 20/80 five years post-op. Those most likely to achieve significant visual improvement (*p* < 0.0001) had concomitant vitreous hemorrhage pre-op. Only 6% of eyes met the US minimum driving standard before surgery based on VA compared to 28% after vitrectomy however this study did not examine visual fields which could warrant additional assessment depending on local requirements. In summary, significant gains in visual acuity are seen after vitrectomy for diabetic TRD that can result in functional improvement in activities of daily living.

## Introduction

Diabetic retinopathy is a leading cause of blindness worldwide^1^, and the top cause of blindness in working-aged adults in most developed countries^2^.Traction retinal detachment (TRD) in the setting of proliferative diabetic retinopathy (PDR) represents one of the most advanced stages of diabetic retinopathy that often requires pars plana vitrectomy (PPV) to restore vision, or prevent further vision loss. Eyes with macula (i.e. fovea) off TRD and combined traction-rhegmatogenous retinal detachment (TRD/RRD) have relatively poor visual outcomes with high rates of re-detachment, as well as progressive vision loss after vitrectomy^3–11^. While there have been recent reports detailing the anatomical successes of modern vitreoretinal surgical instrumentation for diabetic TRDs^12–16^, detailed post-operative visual acuity (VA) changes corresponding to typical daily functions like driving are not well documented.

The World Health Organization (WHO) and International Council of Ophthalmology (ICO) agreed upon three main categories stratifying levels of Snellen VA: near-normal vision (20/12–20/60), low vision (20/80–20/1000), and near-blindness (≤ 20/1000–No Light Perception (NLP))^17–19^. The low vision category is further sub-divided into moderate visual impairment (20/80–20/160), severe visual impairment (20/200 – 20/400), and profound visual impairment (20/500–20/1000)^17^. These categories were chosen to correspond with visual function such that patients with near-normal vision should be able to read without assistive aids whereas patients with ‘low vision’ typically require magnifiers or large-print books^17^. Utilizing this internationally accepted classification scheme allows for estimation of a patient’s function in activities of daily living such as reading or driving.

The most universal requirement for maintaining an unrestricted driver’s license in the United States is based on VA of 20/40 or better in one or both eyes together^20^. Though there are a number of other criteria for a patient to drive^21,22^, determining the ability for an eye to achieve ≥ 20/40 is useful for counseling patients. In the United States, VA loss due to diabetic retinopathy in one eye keeps many commercial drivers (e.g. semi-truck drivers) from working which has a meaningful economic impact between accrued healthcare costs and lost opportunity costs. In the US a VA of ≥ 20/40 is required in each eye to drive with a commercial drivers license. There were 5.7 million commercial motor vehicle operators in the United States in 2012^23^. One study showed that over 7% of commercial drivers have diabetes^24^ which translates to roughly 400,000 affected individuals. According to the 10-year data from the Wisconsin Epidemiologic Study of Diabetic Retinopathy, 16–19% of patients develop PDR and 4–10% have < 20/40 VA^25^. When extrapolated to the number of commercial drivers with diabetes, 64,000–76,000 will develop PDR and 50% will be blind within 5 years^26^. Thus the effect of TRD in those with a commercial drivers license translates into substantive economic impact in America.

We sought to determine the proportion of eyes that sustain significant visual gain (or loss) after vitrectomy for diabetic TRD in those meeting VA-based WHO/ICO ‘low vision’ criteria. We also assessed the number of eyes that attain VA of ≥ 20/40 post-operatively. This study allowed us to relate functional VA outcome surrogates^27^ with long-term anatomic success and complications of patients needing vitrectomy for TRD secondary to PDR in a tertiary, academic hospital setting.

## Methods

This retrospective, non-comparative, interventional, consecutive case series conducted at the University of Iowa followed the guidelines of the Declaration of Helsinki and was approved by University of Iowa Institutional Review Board who deemed that subject consent was unnecessary due to the retrospective nature of the study. Billing codes for TRD, PDR, and vitrectomy were used to formulate a database of patients from 2001–2011; these charts were reviewed for possible entry into the study. The standard principles of TRD repair by vitrectomy were adhered to by all surgeons^28,29^, although individual surgeon preferences on precise technique for different situations could vary. Inclusion criteria were those who had TRD secondary to PDR at the time of vitrectomy. Whether the TRD involved the macula pre-operatively was sometimes difficult to determine as a fair number had VH or lacked detailed records of this. Exclusion criteria were those who had prior PPV. Detailed pre-, intra-, and post-operative data were recorded. Pre-operative data included the patient’s age at the time of surgery, patient’s gender, smoking status, drinking status, type of diabetes, duration of the diabetes, ocular comorbidities, systemic comorbidities, location of the TRD, whether the eye had previous pan-retinal photocoagulation, lens status, best-corrected VA, presence of vitreous hemorrhage (VH), and intraocular pressure. Intra-operative data included gauge of instrumentation used, type of tamponade (i.e. air, gas or oil) employed, adjunctive procedures such as scleral buckle and/or lensectomy, and intra-operative complications. Post-operative data included fellow eye needing vitrectomy, post-op complications, additional surgeries, post-op visual acuity, intra-ocular pressure, and ocular comorbidities. The patient’s VA, intraocular pressure, lens status, and retinal attachment status were recorded at 3, 6, 12, and 60 months post-op, when available. Snellen visual acuities were converted to logMAR scale allowing for statistical analyses. As this was a retrospective study with attrition expected over 60 months of follow-up, VA data were also evaluated for a change of logMAR 0.5 (equivalent to five lines on the ETDRS chart) for those dropping out of study after 3, 6 and 12 months. Patient VA data were stratified based on the WHO classification of ‘near-normal vision’ (> 20/80) and ‘low vision’ (≤ 20/80)^17–19^as well as the universally accepted standard for driving acuity (≥ 20/40)^20^. When evaluating visual recovery, improving three Early Treatment Diabetic Retinopathy Study (ETDRS) lines of vision (logMAR 0.3 equivalent) is most commonly used as an endpoint as this represents a doubling of the visual angle on the ETDRS chart^30^, and has strong statistical significance even at poorer VAs ^31^.

## Results

### General characteristics

A total of 697 charts were found that met the initial search criteria and were reviewed for entry into the study. Seventy one percent (71%) of patients were excluded from analysis because of absence of TRD, absence of DM, absence of PDR, a prior PPV, no PPV, insufficient data, or deviation from the entry time window. A breakdown of this data is in Supplemental Table 1. We analyzed 240 eyes from 203 patients (29% of queried charts) that met all inclusion and exclusion criteria. Thirty-seven patients (18.2%) had PPV in both eyes. At the time of surgery, 215 eyes were phakic (90%) and 25 eyes (10%) were pseudophakic. The mean age at time of surgery was 48 years-old (range 21–88). 106 patients (52%) were male and 97 (48%) female. Twenty-eight eyes (12%) had a combined TRD/RRD. Fifteen eyes (6%) required re-operation for retinal detachment while 3 eyes (1%) ultimately underwent enucleation. Sixty-six eyes, 33.8% of phakic patients post-op, required cataract surgery through the duration of the study with a median post-op time to cataract extraction of 7 months.

**Table 1:** Visual acuity (VA; in logMAR) after vitrectomy for diabetic traction retinal detachment in eyes with pre-operative VA better or worse than 20/80.

Pre-op VA	Post-op time (months)		N	Median logMAR	Lower Quartile logMAR	Upper Quartile logMAR	Min logMAR	Max logMAR	*P* value*
Better than 20/80	3	Pre-op VA (change)	47	0.5 (0.0)	0.3 (− 0.2)	0.5 (0.3)	− 0.1 (− 0.5)	0.5 (3.9)	0.260
	6	Pre-op VA (change)	40	0.5 (0.0)	0.3 (− 0.1)	0.5 (0.2)	− 0.1 (− 0.5)	0.5 (3.9)	0.532
	12	Pre-op VA (change)	34	0.5 (− 0.1)	0.3 (− 0.3)	0.5 (0.3)	− 0.1 (− 0.5)	0.5 (4.9)	0.970
	60	Pre-op VA (change)	13	0.5 (− 0.1)	0.5 (− 0.1)	0.5 (0.0)	0.2 (− 0.2)	0.5 (0.8)	0.429
20/80 or worse	3	Pre-op VA (change)	155	1.8 (− 0.4)	1.0 (− 1.0)	3.3 (0.0)	0.6 (− 3.2)	4.3 (3.7)	<0.0001
	6	Pre-op VA (change)	130	1.8 (− 0.5)	1.1 (− 1.3)	3.3 (0.0)	0.6 (− 3.1)	4.3 (4.2)	<0.0001
	12	Pre-op VA (change)	100	1.8 (− 0.5)	1.1 (− 1.2)	3.3 (0.0)	0.6 (− 3.9)	4.3 (4.2)	0.003
	60	Pre-op VA (change)	44	1.55 (− 0.8)	1.0 (− 0.6)	2.5 (− 0.3)	0.6 (− 0.6)	4.3 (1.0)	0.277

*Wilcoxon signed-rank test.

### Visual outcomes

Median pre-operative VA was 20/320 (n = 240). Post-operative VA was 20/125 at 3 months (n = 202), 20/100 at 6 months (n = 170), 20/100 at 12 months (n = 134), and 20/80 at 60 months (n = 57). The changes in VA from pre-op were compared at 3, 6, and 12 months post-op follow-up in these groups (demonstrated in Table 1) with data extending to 60 months.

**Figure 1:**
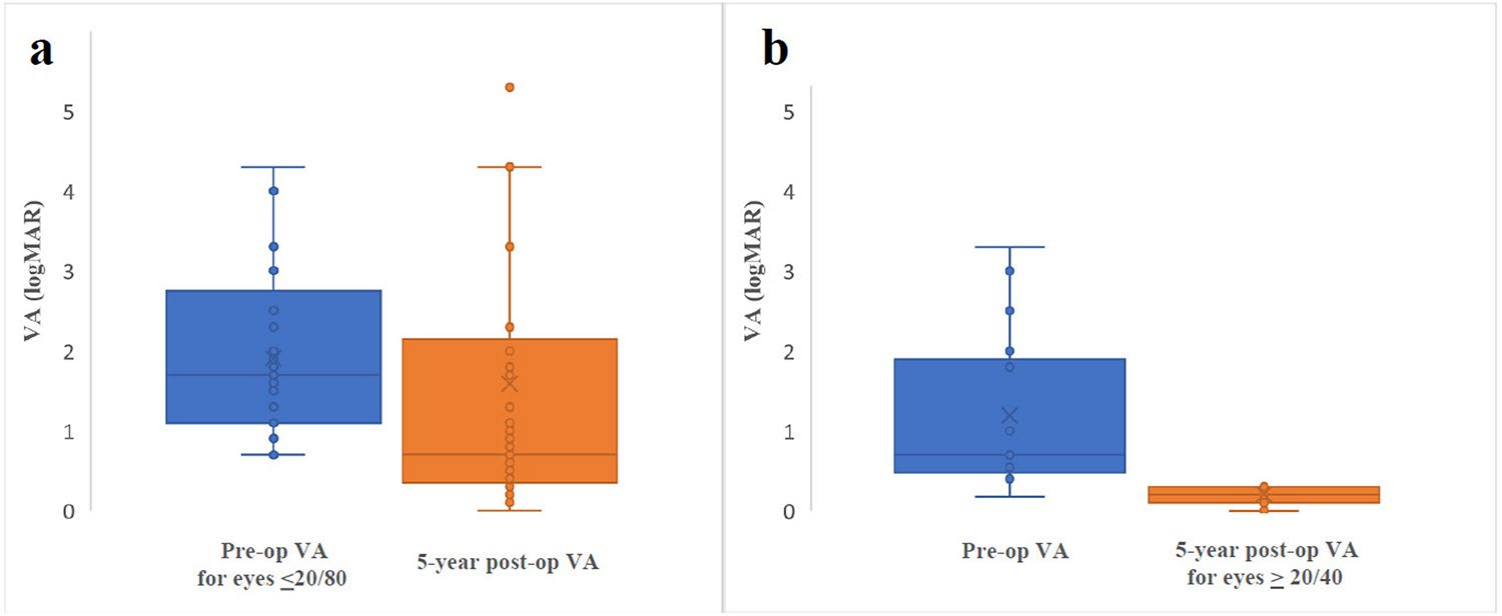
Patients who underwent vitrectomy with traction retinal detachment with a) pre-op visual acuity (VA) compared to 5 years after surgery for patients with pre-op vision of ≤ 20/80. Mean pre-op VA was 1.9 logMAR (Snellen equivalent 20/1600); 5 years post-op was 1.6 logMAR (Snellen equivalent 20/800) and b) pre-op visual acuity (VA) compared to 5 years after surgery for patients with post-op vision of ≥ 20/40. Mean pre-op VA was 1.2 logMAR (Snellen equivalent 20/300); 5 years post-op was 0.2 logMAR (Snellen equivalent 20/30).

Pre-operatively, 79% (190/240) of eyes had VA ≤ 20/80. At 12 months, 57% of eyes with a pre-op VA ≤ 20/80 improved 0.3 logMAR, whereas 26% of those with a pre-op VA of > 20/80 improved 0.3 logMAR (Table 2). At 60 months of follow up, there was no statistically significant difference in VA for those patients with pre-op VA ≤ 20/80, *p* = 0.16 (Fig. 1). Stability in VA (< 0.3 logMAR change) was seen in 47% of eyes with pre-op VA of > 20/80 at 12 months (Table 2). Patients with VH pre-operatively tended to have greater improvement in VA compared to those without pre-operative VH. Among patients who had ≤ 20/80 VA and a VH at the time of operation, the median logMAR change in visual acuity was -0.6 at three months (n = 119; *p* < 0.0001), − 0.7 at 6 months (n = 100; *p* < 0.0001), and -0.7 at 12 months (n = 75; *p* < 0.0001). In patients with ≤ 20/80 VA without a VH, no significant change in vision was noted at the different post-operative time points. In patients with > 20/80 VA there was no significant change in vision at the various post-operative time points regardless of presence or absence of VH.

**Table 2:** Percentage of eyes with change in visual acuity (VA) of at least 0.3 logMAR after vitrectomy for diabetic traction retinal detachment when pre-operative VA was better or worse than 20/80.

Pre-op VA	Post-op time (months)	N	Improve [ > 0.3 increase]	Same [< 0.3 change]	Worse [> 0.3 decrease]
Better than 20/80	3	47	7 (15%)	28 (60%)	12 (26%)
	6	40	5 (12%)	28 (70%)	7 (18%)
	12	34	9 (26%)	16 (47%)	9 (26%)
	60	13	2 (15%)	9 (70%)	2 (15%)
20/80 or worse	3	155	83 (54%)	43 (28%)	29 (19%)
	6	130	79 (61%)	24 (18%)	27 (21%)
	12	100	57 (57%)	23 (23%)	20 (20%)
	60	44	26 (59%)	8 (18%)	10 (23%)

While the pre-operative percentage of eyes with VA ≥ 20/40 was just 6% (15/240), 28% (37/134) of eyes had VA ≥ 20/40 at 12 months post-op. The percentage of eyes with VA ≥ 20/40 held steady at 30% (29/95) at 24 months and 28% (16/57) at 60 months post-op. A substantial change occurred in eyes in the near-normal vision category as 21% (50/240) with VA > 20/80 pre-operatively increased to 43% (58/134) at 12 months, 43% (41/96) at 24 months, and 49% (28/57) at 60 months (Fig. 1). Nearly 20% of eyes with pre-op VA of ≤ 20/80 achieved VA of ≥ 20/40 after vitrectomy (Table 3).

**Table 3:** Percentage of eyes with post-operative visual acuity (VA) ≥ 20/40 and > 20/80 when pre-operative VA was better or worse than 20/80.

Pre-op VA	Post-op time (months)	N	Post-op VA ≥ 20/40	Post-op VA > 20/80
Better than 20/80	3	47	20 (43%)	30 (64%)
	6	40	18 (45%)	29 (73%)
	12	34	18 (53%)	25 (74%)
	60	13	7 (54%)	11 (85%)
20/80 or worse	3	155	28 (18%)	44 (26%)
	6	130	23 (18%)	45 (35%)
	12	100	19 (19%)	33 (33%)
	60	44	17 (32%)	28 (64%)

We found no statistically significant association for age, gender, tamponade agent, tobacco use, lens status, or intra-op complications in those who had improvement in visual acuity of at least 0.3 logMAR at 12 months with initial VA ≤ 20/80 (Table 4). Demographics for those presenting at 3 months follow up are shown in Supplemental Table 2. No statistically significant difference in visual outcomes was identified between 20-gauge compared to 23 combined with 25-gauge vitrectomies at any time point, although a small trend of 0.1 – 0.2 logMAR gains in visual acuity was seen in the 23/25-gauge group at each time point compared to 20 gauge.

**Table 4:** Association of patient factors with improvement in visual acuity of at least 0.3 logMAR at 12 months in those with initial VA of 20/80 or worse.

Variable	Improved VA Count (%)	*p* value
**Gender**		
Female n = 51	27 (53%)	0.403^+^
Male n = 49	30 (61%)	
**Current smoker**		
Yes n = 19	11 (58%)	0.892^+^
No n = 73	41 (56%)	
**Drink alcohol**		
Yes n = 17	10 (59%)	0.789^+^
No n = 76	42 (55%)	
**Tamponade**		
C3F8 n = 25	13 (52%)	0.222^+^
SF6 n = 21	15 (71%)	
SO n = 14	5 (36%)	
Air n = 5	4 (80%)	
None n = 35	20 (57%)	
**Lens status**		
Phakic n = 56	29 (52%)	0.235^+^
Pseudophakic n = 44	28 (64%)	
**Intra-op complications**		
Yes n = 21	10 (48%)	0.329^+^
No n = 79	47 (59%)	
**Age Mean(SD)**		
Improved VA (n = 57)	47.7 (13.0)	0.492#
Same/Worse VA (n = 43)	49.6 (14.8)	

^+^Pearson Chi-square; ^#^ t-test.

### Effect of attrition on visual outcomes

Since attrition rate may skew outcomes in this study (n = 240 pre-operatively; n = 202 at 3 months, n = 170 at 6 months, n = 134 at 12 months, and n = 57 at 60 months), we compared available post-operative acuities among patients who dropped out at 6 and 12 months. There was no significant change in the percentage of patients who stayed the same (logMAR change of < 0.5), worsened (increase of 0.5 in logMAR acuity), or improved (decrease of 0.5 in logMAR acuity) at the three-month visit, regardless of attrition. Similarly, visual outcomes at 6 months were not significantly different among those who dropped out at 12 months and those who did not.

### Characteristics of patients with poor outcome

Twenty-four eyes (10%) had light perception or worse visual acuity post-operatively. The 23 patients who perceived NLP or LP vision two years post-operatively had an average pre-op vision that was significantly worse compared to those who did not develop such vision (3.6 logMAR acuity vs 1.5 logMAR acuity, *p* < 0.0001). Using average pre-op logMAR acuity of 4.3 (LP) as a predictor for poor visual outcome, we evaluated the performance of such a cut-off in predicting poor surgical outcomes in our data set. While such a cut off is specific (94.6%) for predicting poor outcomes (defined as LP or NLP vision by two years post-operatively) the sensitivity and positive predictive value of such a cut-off is very poor (26.4 and 27.5%, respectively). Thirteen eyes developed NLP vision, all initially presenting with either LP or HM pre-op vision. Eyes became NLP due to a combination of mechanisms including neovascular glaucoma, closed funnel RD, ischemic retinopathy/optic neuropathy, and phthisis bulbi. A percentage of patients with poor visual acuity at two years had the following characteristics identified pre-operatively: TRD involving the fovea (45.8%), four had star folds (16.7%), three had macular holes (12.5%), and three had breaks outside the area of TRD (12.5%).

Eight patients underwent surgery who had LP vision pre-operatively. Average logMAR acuity post-operatively at 1 year was 4.1 logMAR (4.3 logMAR = light perception vision). There was no significant difference in VA pre- vs. post-operatively in this group (*p* = 0.5165, n = 8). Sixty-three percent of these patients either became worse or stayed light perception vision, with the remaining achieving counting fingers vision (n = 1) or hand motion vision (n = 2).

## Discussion

This study shows that before surgery nearly 80% of eyes with TRD from PDR have low vision; after vitrectomy over half of these eyes had significant improvement in visual acuity, corresponding with functional improvements based on visual acuity. Pre-operatively only 6% of eyes with TRD from PDR achieved a VA of ≥ 20/40, while almost 30% of eyes had VA ≥ 20/40 post-operatively with 5 years of follow-up. Eyes with VH were more likely to obtain statistically significant gains in VA. These data point to the real world, functional value of vitrectomy for TRD from PDR that can give valuable information to physicians, patients and their support systems on long-term expectations after surgery.

TRD from PDR is a challenging condition to manage due to often complex vitreoretinal traction, retinal ischemia, neovascularization and relatively poor prognosis with or without surgery. Recent data shows 50% of patients with TRD and non-clearing VH, and 87% of patients with non-clearing VH alone, have at least a three ETDRS line improvement one year after vitrectomy surgery^32^. Another study found that approximately 80% of eyes have improved or stabilized vision after TRD repair with similar outcomes between PPV gauges^15^. This study supports these findings but specifically evaluated the outcomes based on the internationally accepted cut-off for ‘low vision’ (≤ 20/80) established by the WHO and the ICO^17^. It also determined how often patients regained the widely accepted minimum VA for an unrestricted US drivers license, ≥ 20/40, according to the U.S. Department of Transportation and the National Highway Traffic Safety Administration^33^. These results are consistent with previously published data^9,34^. As discussed, these surgical outcomes have a large socioeconomic impact on the country with respect to commercial drivers. There are other factors that could limit driving function in patients with PDR beyond loss of VA such as loss of peripheral field from PRP^21,22,35,36^; a limitation to this study is that we did not include assessment of VFQ-25 or visual fields. We also did not analyze the status of the fellow eye, which may or may not have been good enough to meet the vision minimum for driving.

There was no improvement in VA seen in patients with a pre-op VA of light perception (though the number with eyes with LP VA pre-op was small), suggesting that it is severe, irreversible ischemic retinopathy and/or optic neuropathy that limits our ability to help patients with the most advanced disease. A second PPV was required in 6% of eyes compared to 10% in other studies and the rate of post-operative vision of LP or worse was 10% which is similar or favorable to other studies^15,37^. When looking at visual outcomes between different gauge surgeries, no difference in visual outcomes was found when comparing 20 gauge to 23 and 25 gauge systems. In both groups 18%-26% of patients had worse vision than prior to surgery. These results are commensurate with a recently published large data set by Storey et al. where 80% of eyes showed stable or improved vision and no difference in outcomes detected between different gauge systems^15^. Notably, there are limitations with using logMAR 0.3 of improvement of visual acuity as a statistically significant standard despite widespread acceptance and use^31^.

This study evaluated a patient cohort cared for by several different vitreoretinal surgeons with the goal of approximating typical outcomes from a single academic center. This cohort is comprised of primarily caucasian patients which may not extrapolate well to other areas of the world. There was moderate attrition throughout the length of follow-up, but this was partly due to the nature of a tertiary referral center whereby patients are routinely discharged to follow locally as soon as is medically appropriate (often occurring around 3 months post-op).

Ultimately, these data provide the ability to appropriately counsel diabetic patients needing surgery for TRD that visual acuity gains typically occur within 6–12 months after surgery and are maintained out to 5 years. Around 30% of all patients achieve visual acuity good enough to drive and over 50% of all patients achieve near-normal vision correlating with improved function in activities of daily living. This enables a better understanding of what can be expected from modern vitreoretinal surgery for such a challenging condition and how classification of visual outcomes can correlate with patient functional outcomes.

## Supplementary information

Supplementary file1

Supplementary file2
