# Supplementary material for: Attaining functional levels of visual acuity after vitrectomy for retinal detachment secondary to proliferative diabetic retinopathy
Source: Sci Rep. 2020 Sep 24;10:15637. doi: 10.1038/s41598-020-72618-y (PMC7519031; doi:10.1038/s41598-020-72618-y)
Supplement: Supplementary file 1 — Supplementary file1 [file 41598_2020_72618_MOESM1_ESM.pdf]

Attaining functional levels of visual acuity after vitrectomy for retinal detachment secondary to proliferative diabetic retinopathy

Aaron Ricca, MD, Kiley Boone, BA, H. Culver Boldt, MD, Karen M. Gehrs, MD, Stephen R. Russell, MD, James C. Folk, MD, M. Bridget Zimmerman, PhD, Mark E. Wilkinson, OD, Elliott H. Sohn, MD

Supplemental table 1. Number of patients included and reasons for exclusion

Total patients in initial broad search	697
Reasons for exclusion	
Insufficient data	11
No pars plana vitrectomy (PPV)	40
Wrong time frame	14
Previous PPV in same eye	18
No diabetes mellitus	182
No proliferative diabetic retinopathy	12
No traction retinal detachment present at time of vitrectomy	217
Total patients excluded	494
Total number of patients meeting inclusion and exclusion criteria	203
Total number of eyes meeting inclusion and exclusion criteria	240
