# Supplementary material for: Attaining functional levels of visual acuity after vitrectomy for retinal detachment secondary to proliferative diabetic retinopathy
Source: Sci Rep. 2020 Sep 24;10:15637. doi: 10.1038/s41598-020-72618-y (PMC7519031; doi:10.1038/s41598-020-72618-y)
Supplement: Supplementary file 2 — Supplementary file2 [file 41598_2020_72618_MOESM2_ESM.pdf]

Attaining functional levels of visual acuity after vitrectomy for retinal detachment secondary to proliferative diabetic retinopathy

Aaron Ricca, MD, Kiley Boone, BA, H. Culver Boldt, MD, Karen M. Gehrs, MD, Stephen R. Russell, MD, James C. Folk, MD,
M. Bridget Zimmerman, PhD, Mark E. Wilkinson, OD, Elliott H. Sohn, MD

Supplemental table 2. Patient demographics of those who presented for 3 month follow-up.

	**N (eyes)**	**%**
Gender		
Male	106	52
Female	96	48
Tamponade agent used at time of surgery		
C3F8	45	22
SF6	43	21
Air	26	13
No tamponade	68	34
Silicone Oil	19	9
Vitreous hemorrhage		
Present	151	77
Absent	45	23
Lens status		
Phakic	180	90
Pseudophakic	20	10
Smoking status		
Smoker	38	20
Non-smoker	155	80
Laterality		
OD	106	44
OS	134	56
1 or 2 operated eyes		
Single	166	82
Both	37	18
Presence of RRD		
TRD alone	175	88

TRD/RRD combined	28	12
